# Proteomic Analysis of the Protective Effect of Eriodictyol on Benzo(a)pyrene-Induced Caco-2 Cytotoxicity

**DOI:** 10.3389/fnut.2022.839364

**Published:** 2022-03-03

**Authors:** Chong Wang, Fan Zhao, Yun Bai, Chunbao Li, Xinglian Xu, Karsten Kristiansen, Guanghong Zhou

**Affiliations:** ^1^College of Food Science and Technology, Nanjing Agricultural University, Key Laboratory of Meat Products Processing, Ministry of Agriculture, Jiangsu Collaborative Innovation Center of Meat Production and Processing, Quality and Safety Control, Nanjing, China; ^2^Laboratory of Genomics and Molecular Biomedicine, Department of Biology, University of Copenhagen, Copenhagen, Denmark; ^3^BGI-Shenzhen, Shenzhen, China; ^4^Institute of Metagenomics, Qingdao-Europe Advanced Institute for Life Sciences, BGI-Qingdao, Qingdao, China

**Keywords:** benzo(a)pyrene, eriodictyol, Caco-2 cells, cytotoxicity, proteomics

## Abstract

We evaluated the possible protective effects of six polyphenols on benzo(a)pyrene (BaP)-induced cytotoxicity in Caco-2 cells. We show that treatment with quinic acid, ferulic acid, homovanillic acid, trolox and BaP decreased cell viability, whereas naringenin and eriodictyol affected viability in a bi-phasic manner with low concentrations decreasing viability whereas higher concentrations increase viability. Co-treatment with 20 μM eriodictyol or naringenin reduced BaP-induced cytotoxicity, including cell apoptosis, cell cycle progression, and oxidative stress. Our results show that the protective effect of eriodictyol was superior to that of naringenin. The potential protective mechanisms of eriodictyol on BaP-induced toxicity were investigated by proteomics. We identified 80 differentially expressed proteins (DEPs) with proteins associated with genetic information processing pathway representing the highest proportion and number of proteins responding to eriodictyol treatment, including key proteins such as RPA2, SNRPA, RAD23B, NUP155 and AARS. Our results provide new knowledge on how polyphenols may prevent BaP-induced carcinogenesis.

## Introduction

Benzo(a)pyrene (BaP), a well-known genotoxic polycyclic aromatic hydrocarbon (PAH), is a ubiquitous environmental chemical carcinogen, mainly present in cigarette smoke, incompletely combusted crude oils, coal tars, and certain types of processed foods. Humans are exposed to BaP by inhalation and intake, particularly *via* food which contributes with 97% of the intake. Long-term exposure to BaP can elicit genotoxic, neurotoxic, mutagenic and carcinogenic responses in various organs and tissues (1). As part of its carcinogenic mechanism, BaP is activated by phase I and II metabolizing enzymes generating 7, 8-diol-9, 10-epoxide (BPDE), which may interfere with cellular processes by covalently binding to DNA, eventually linked to carcinogenesis (2). In addition, BaP is associated with formation of reactive oxygen species (ROS) that may induce the generation of the highly reactive genotoxic BaP-quinone component, 8-hydroxy-2-deoxyguanosine, 8-oxo-dG (3).

BaP is generated during the preparation of food such as grilling, frying, and roasting. These processes significantly increase the content and accumulation of BaP in the food. The use of herb and dietary supplements such as polyphenols to protect against the detrimental effects of BaP has gained support worldwide. Many studies have shown that polyphenols hold promises for reducing DNA damage, oxidative stress, and carcinogenesis induced by BaP in *vitro* and *in vivo* (4, 5). In *in vitro* studies, primary cultured neurons (6), HepG2 cells (7) and Bhas 42 cells (8) have been used to investigate how polyphenols may counteract the detrimental effects of BaP exposure. In addition, several animal studies have reported that oral administration of polyphenols such as quercetin (9), curcumin (10), and galangin (11) elicited protection against BaP-induced damage of the lung and other organs.

Reducing the production of BaP is another important way to prevent exposure. Zhao et al. (12) reported on a 71.75 and 74.80% reduction in PAHs and oxygenated PAHs (OPAHs), respectively, when tert-butylhydroquinone (TBHQ) was added to the frying oil. This reduction in the formation of PAHs and OPAHs can be attributed to the antioxidant properties of TBHQ (12). Analysis of the effect of synthetic [e.g., butylated hydroxyanisole (BHA) and 3,5-di-tert-4-butylhydroxytoluene (BHT)] and natural (e.g., epigallocatechin gallate (EGCG), α-tocopherol, and sesamol) antioxidants on PAH generation in heated meat model systems revealed that total PAHs decreased upon addition of antioxidants (13). Our previous studies have shown that polyphenols such as eriodictyol, naringenin, quinic acid, ferulic acid, homovanillic acid, and trolox in tea and beer effectively can inhibit the production of BaP in chicken wings during grilling (14, 15). However, whether these polyphenols can also reduce the toxicity of BaP at the cellular level, and the mechanisms behind such a possible action have not yet been studied.

High-throughput, proteomics has become a powerful approach for understanding the mechanisms of toxicity and for the development of specific biomarkers for BaP exposure. Proteome profiles comparing BaP-transformed and normal 16HBE cells have revealed that FOXA1 is a key protein in BaP-induced lung cancer *via* its ability to increase colony formation and migration *in vitro*, and promote tumor growth and metastasis *in vivo* (16). Proteomics studies have identified a substantial number of candidate proteins associated with BaP toxicity. However, few of these proteins have been investigated in order to decipher their biological functions in BaP-induced colonic cancer. Therefore, studies to identify key proteins understanding their biological role in BaP-induced carcinogenesis, and determine how polyphenols may prevent or alleviate the detrimental effects of BaP are still warranted.

The intestine plays a critical role in the primary defense against carcinogens and toxic compounds, and the human colon carcinoma cell line Caco-2 has been widely used in studies of the actions of xenobiotics (17). In this study, we evaluated whether polyphenols (eriodictyol, naringenin, quinic acid, ferulic acid, homovanillic acid and trolox) are critical for reducing BaP-induced cell damage. We used proteomics to investigate possible mechanisms by which eriodictyol might alleviate BaP toxicity in Caco-2 cells.

## Materials and Methods

Benzo(a)pyrene (BaP, ≥ 96% HPLC), eriodictyol (≥ 95% HPLC), naringenin (≥ 95% HPLC), quinic acid (≥ 98% HPLC), ferulic acid (≥ 99% HPLC), homovanillic acid (≥ 95% HPLC), trolox (≥ 98% HPLC), dimethyl sulfoxide (DMSO), glacial acetic acid, thiobarbituric acid (TBA), Tris-HCl, dithiothreitol (DTT), iodoacetamide, ammonium bicarbonate, formic acid, acetonitrile, urea were obtained from Sigma-Aldrich Chemical (St. Louis, MO, USA). Protease inhibitor cocktail, phosphatase inhibitor cocktail, BCA protein assay kit, RIPA lysis and extraction buffer, Dulbecco modified Eagle medium (DMEM), fetal bovine serum (FBS), penicillin-streptomycin, trypsin, phosphate-buffered saline (PBS), cell apoptosis kit, cell cycle assay kit were obtained from Thermo Fisher Scientific (Waltham, MA, USA). Sequencing-grade trypsin was obtained from Promega (Madison, WA, USA). Superoxide dismutase (SOD) assay kit and malondialdehyde (MDA) assay kit were obtained from JianCheng Bioengineering Institute (Nanjing, China). Antibodies (RPA2, SNRPA, RAD23B, NUP155 and AARS) and HRP-conjugated anti-rabbit immunoglobulin G (IgG), were purchased from Sigma-Aldrich Chemical (St. Louis, MO, USA).

### Cell Culture and Treatments

The human colon carcinoma cell line Caco-2 was obtained from Jiangsu KeyGen BioTech (Nanjing, China). Caco-2 cells were grown in DMEM containing 10% FBS and 1% penicillin–streptomycin. The incubator was kept at 37°C with 5% CO_2_ and the medium was replaced every 2 days until confluence reached 80%.

The trial was set up including the following three parts. In the first part, cells were treated with BaP (1, 2, 5, 10, 20, 50 and 100 μM) for different times (3, 6, 12, 24 and 48 h) and 6 types of polyphenols (1, 2, 5, 10 and 20 μM) for 24 h to evaluate the cytotoxicity of BaP and polyphenols. In the second part, cells were Pre-treated or co-treated with eriodictyol (5, 10 and 20 μM), naringenin (20 μM) and BaP (50 μM) for 24 h to assess the protective effects of the polyphenols on BaP-induced cytotoxicity. In the third part, cells were co-treated with eriodictyol (20 μM) and BaP (50 μM) to elucidate the possible mechanism by which eriodictyol may counteract the detrimental effects of BaP using proteomics. The doses of BaP used to treat the cells were selected according to previous *in vitro* studies and do not reflect the amounts attainable by oral intake.

### Cell Viability Assay

Cell viability was measured as previously described with slight modifications (18). MTT was dissolved in dilution buffer at a final concentration of 5 mg/mL. After experimental treatments, MTT solution (10 μL) was added to each well. After incubation at 37°C for 4 h, the formed formazan crystals were dissolved in 1 mL of DMSO and the absorbance was measured at 570 nm. The results were calculated as following:


$$\begin{array}{l} \text{Cell viability }\left(\%\right)=\frac{\text{average OD of treatment}}{\text{average OD of control}}\times 100\% \end{array}$$

### Cell Apoptosis and Cycle Assay by Flow Cytometry

Cell apoptosis was assessed using annexin as the marker according to the manufacturer's recommendations. Briefly, the cells were washed with PBS and harvested with trypsin. After centrifugation at 800 rpm for 5 min, cells were resuspended in 0.5 mL binding buffer. 5 μL Annexin-V EGFP and 5 μL propidium iodide (PI) were added. The tubes were incubated for 20 min in the dark at room temperature. Cells were measured by flow cytometry within 1 h. For cell cycle analysis, cells were collected and fixed in 70% ethanol overnight at 4°C. Cells were washed with PBS three times, and 25 μL PI and 10 μL RNase A (50 μg/mL) were added. The tubes were incubated at 37°C for 30 min. The excitation and emission wavelengths were 488 nm and 530 nm, respectively. The Annexin-V EGFP and PI channel was set as FL1-A and FL2-A, respectively.

### ROS, SOD Activity and MDA Content

Intracellular ROS was measured using the DCFH-DA method (19). After treatments, cells were washed with PBS and then treated with DCFH-DA (10 μM) for 30 min. Subsequently, cells were collected and washed with PBS two times. ROS-dependent fluorescence was detected using a Leica DMI 6000B (Wetzlar, Germany), and then the cells were transferred into a black 96-well plate to detect fluorescence intensity using a Multimode Reader (TECAN, Switzerland). The excitation and emission wavelengths were 485 and 535 nm, respectively. SOD activity and MDA content were assessed according to the manufacturer's protocol. In addition, the protein content was measured using the BCA protein assay kit. The enzyme activity and MDA content were expressed as units per mg of protein (U/mg protein) and μM per mg of protein (μM/mg protein).

### Protein Digestion

Protein samples (200 μg) were digested using the filter-aided sample preparation (FASP) method (20). In brief, protein was reduced with 10 mM DTT for 1 h at 60°C, alkylated in the presence of 55 mM of iodoacetamide for 45 min at 25°C in the dark. Subsequently, the buffer was exchanged with 100 mM ammonium bicarbonate (pH 8.5) using a 10 kDa molecular weight cut-off ultrafiltration tube (Millipore, Billerica, MA, USA). After that, 4 μg of trypsin were added to each sample for protein digestion overnight at 37°C (trypsin: protein, 1: 50 w/w). The digested peptides were desalted using Sep-Pak C18 cartridges (Waters, Milford, USA) and quantified using a NanoDrop spectrophotometer at 280 nm.

### Proteomics Analysis by LC-MS

A Nano-LC tandem with a linear trap quadrupole mass spectrometer (Thermo Fisher Scientific, USA) was applied to analyze the protein profiles. The resulting peptides (1.5 μg) were acidified with 0.1% formic acid and subsequently loaded onto the C18 column (75 μm × 15 cm, 3 μm, 100 Å; Thermo-Fisher Scientific). Chromatographic separation was carried out with a linear gradient of 3–55% buffer B (80% acetonitrile and 0.1% FA) at a flow rate of 0.25 μL/min over 112 min. Due to loading and washing steps, the total time for an LC-MS/MS run was ~160 min. Electrospray ionization (ESI) was applied in the positive mode with the following parameters: MS data were acquired using a data-dependent top 10 method dynamically exclusion to screen the most abundant precursor ions from the survey scan (300–1800 m/z) for HCD fragmentation. Dynamic exclusion duration was 25 s. Survey scans were acquired at a resolution of 70,000 at m/z 200 and the resolution for HCD spectra was set to 17,500 at m/z 200.

A label-free method was applied for protein quantification. The MS data were analyzed using the MaxQuant software (version 1.3.0.5) and searched against the corresponding UniProt *Homo sapiens* database. The precursor mass and MS/MS tolerance of peptides were set to 6 and 20 ppm, respectively. The maximum number of missed cleavages was two. The carbamidomethylation of cysteine was set as a fixed modification, with protein *N*-terminal oxidation of methionine as a variable modification. The false discovery rate (FDR) was set to 1%. Protein abundance was calculated on the basis of the normalized spectral protein intensity (LFQ intensity). Differentially expressed proteins (DEPs) were characterized as proteins with a fold change in intensity > 1.5 or <0.67 and *p* < 0.05. The Kyoto Encyclopedia of Genes and Genomes (KEGG) and protein-protein interaction analysis were performed using Omicsbean (http://www.omicsbean.cn). The strengths of the PPI network relationships were visualized by assigning line weights to the compiled scores. PPI analysis was done with minimum required interaction score set to medium confidence 0.400.

### Western Blotting

The protein sample was mixed with loading buffer and heated at 95°C for 5 min. 20 μg samples and standard protein (Bio-Rad, Hercules, CA, USA) were loaded on a gradient polyacrylamide gel (4–10%, Genscript, Piscataway, USA). The gel was run at 110 V for 120 min (4°C) and then the proteins were transferred onto a polyvinylidene difluoride membrane. After transfer at 120 V for 90 min, the membrane was blocked in 5% bovine serum albumin for 60 min at room temperature. Subsequently, the membrane was incubated with the primary antibodies overnight at 4°C and then incubated with anti-rabbit IgG for 60 min. The detection was preformed using a chemiluminescence system (Thermo Fisher Scientific, Rockford, IL, USA), and the bands were analyzed using the Quantity One system (Version 4.6.2).

### Statistical Analysis

All results were calculated as the means with standard deviations. The results were statistically analyzed by ANOVA (*p* < 0.05). Comparison of mean values was performed using Duncan's test. Statistical analyses were performed with SPSS for Windows version 20 (SPSS Inc., Chicago, IL).

## Results and Discussion

### Cytotoxicity of BaP and Polyphenols on Caco-2 Cells

**Figure 1:**
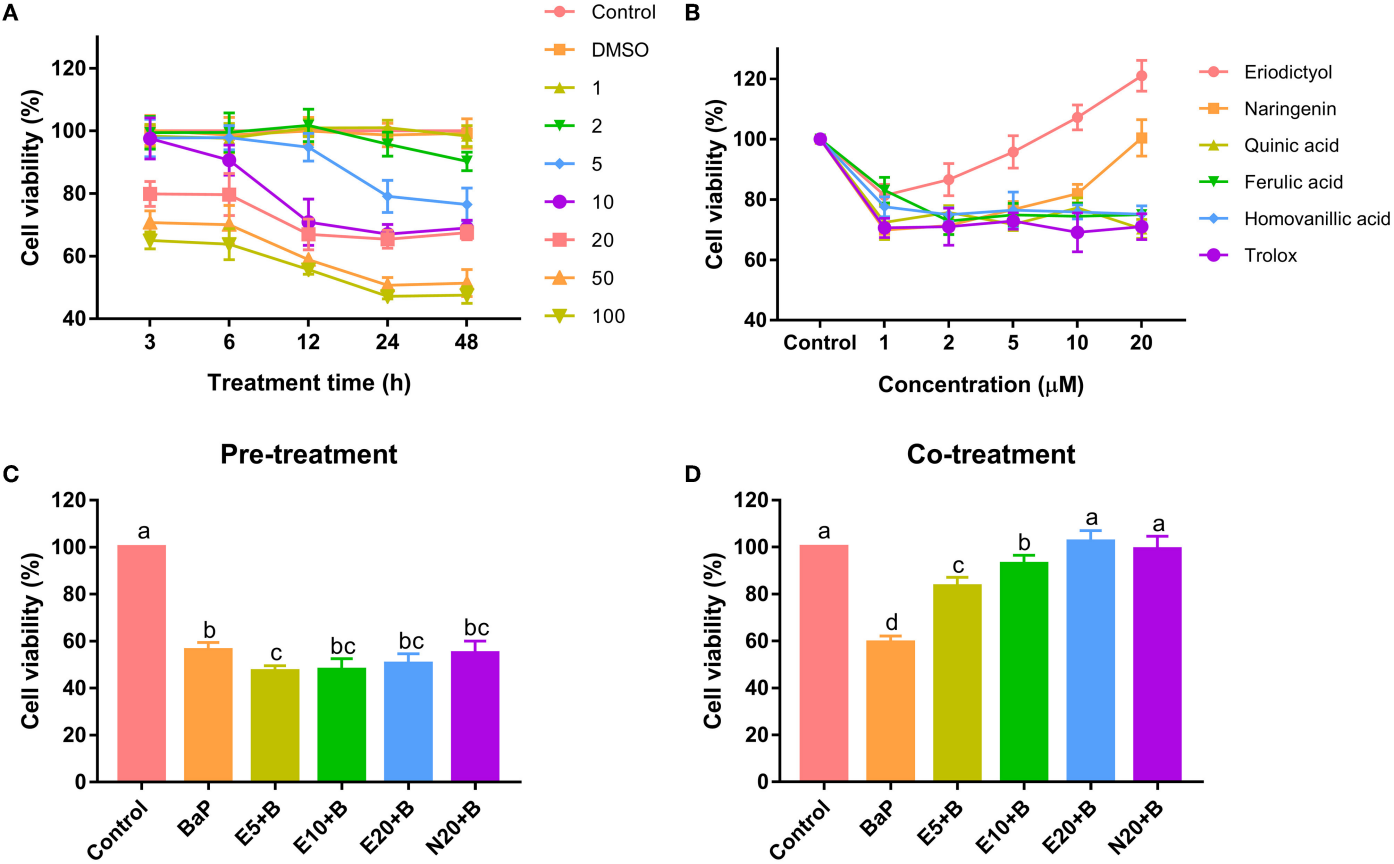
Cytotoxicity of benzo(a)pyrene and polyphenols on Caco-2 cells. Caco-2 cells were incubated with different concentrations of benzo(a)pyrene **(A)**, polyphenols **(B)**, Pre-treatment with 5, 10, 20 μM eriodictyol or 20 μM naringenin **(C)** with 50 μM BaP, co-treatment with 5, 10, 20 μM eriodictyol and 20 μM naringenin **(D)** with 50 μM BaP. Cell viability was measured by the MTT method. Results are expressed as percentage of control values. Error bars represent the standard deviation obtained from three replicated experiments. Bars with different letters are significantly different at the level *p* < 0.05. E refers to eriodictyol, *N* refers to naringenin and B refers to BaP.

Caco-2 cells were treated with different doses for different period of time with BaP and polyphenols to define the most suitable conditions for the exposure to BaP and polyphenols. BaP significantly decreased cell viability in a dose- and time-dependent manner in concentrations ranging from 2 to 100 μM **(Figure 1A)**. Treatments with 50 μM BaP for 24 and 48 h significantly decreased cell viability by 50.7 and 51.4%, respectively. At any treatment time, there was no significant difference in cell viability between the 50 and 100 μM treatment groups. As shown in previous studies, BaP significantly inhibited cell viability of HepG2 cells (7), HL-7702 cells (21) and HELF cells (22). The concentrations of BaP that caused a decrease in cell viability are within the range reported in other studies (21, 23).

The cells were treated with different concentrations of polyphenols to investigate possible effects on viability or cytotoxicity. A decrease in viability was observed when Caco-2 cells were treated with quinic acid, ferulic acid, homovanillic acid and trolox at any concentration for 24 h (Figure 1B). This result is in accordance with previous studies showing that cell viability is reduced in a concentration-dependent manner when cells were treated with quinic acid, ferulic acid, or homovanillic acid (24, 25). Interestingly, treatment with eriodictyol and naringenin resulted in a reduction in cell viability at low concentrations, for naringenin from 1 to 10 μM, for eriodictyol at concentrations of 1 and 2 μM. However, treatment with eriodictyol at concentrations of 5, 10 and 20 μM for 24 h or treatment with naringenin at 20 μM revealed no toxicity, and for eriodictyol, treatment even appeared to increase the apparent cell viability at high concentrations (10 and 20 μM), suggesting increased cell proliferation. Based on the results, we used 50 μM BaP and Non-toxic concentration eriodictyol (5, 10 and 20 μM) and naringenin (20 μM) for the following exposure experiments with mixtures of the compounds.

To evaluate the protective effect of eriodictyol and naringenin on BaP-induced cytotoxicity, cells were co-treated with the polyphenols or pre-treated with the polyphenols prior to administration of BaP. Pre-treatment with eriodictyol and naringenin was unable to alleviate the toxicity of BaP in the cell viability assay (Figure 1C). However, co-treatment with 20 μM eriodictyol or naringenin completely restored viability (Figure 1D). It is known that BaP displays its toxicity after modification by P450 monooxygenase to generate a series of metabolites which react with DNA. Polyphenols can form adducts with BaP and its metabolites, which reduces the bioavailability of BaP (26). In the co-treatment group, we speculate that both eriodictyol and naringenin formed complexes with BaP and its metabolites. However, in the pre-treatment group, most of the polyphenols may have been completely metabolized after treatment for 24 h abolishing their effect on BaP. A similar observation was reported for fresh cashew apple juice where co- and post-treatment showed a reduced mutagenic effect of BaP, while pre-treatment had no effect (27). Based on the cell viability data, 20 μM of eriodictyol and naringenin were used for further experiments.

### Effect of Eriodictyol and Naringenin on BaP-Induced Cell Cycle Perturbation and Cell Apoptosis

**Figure 2:**
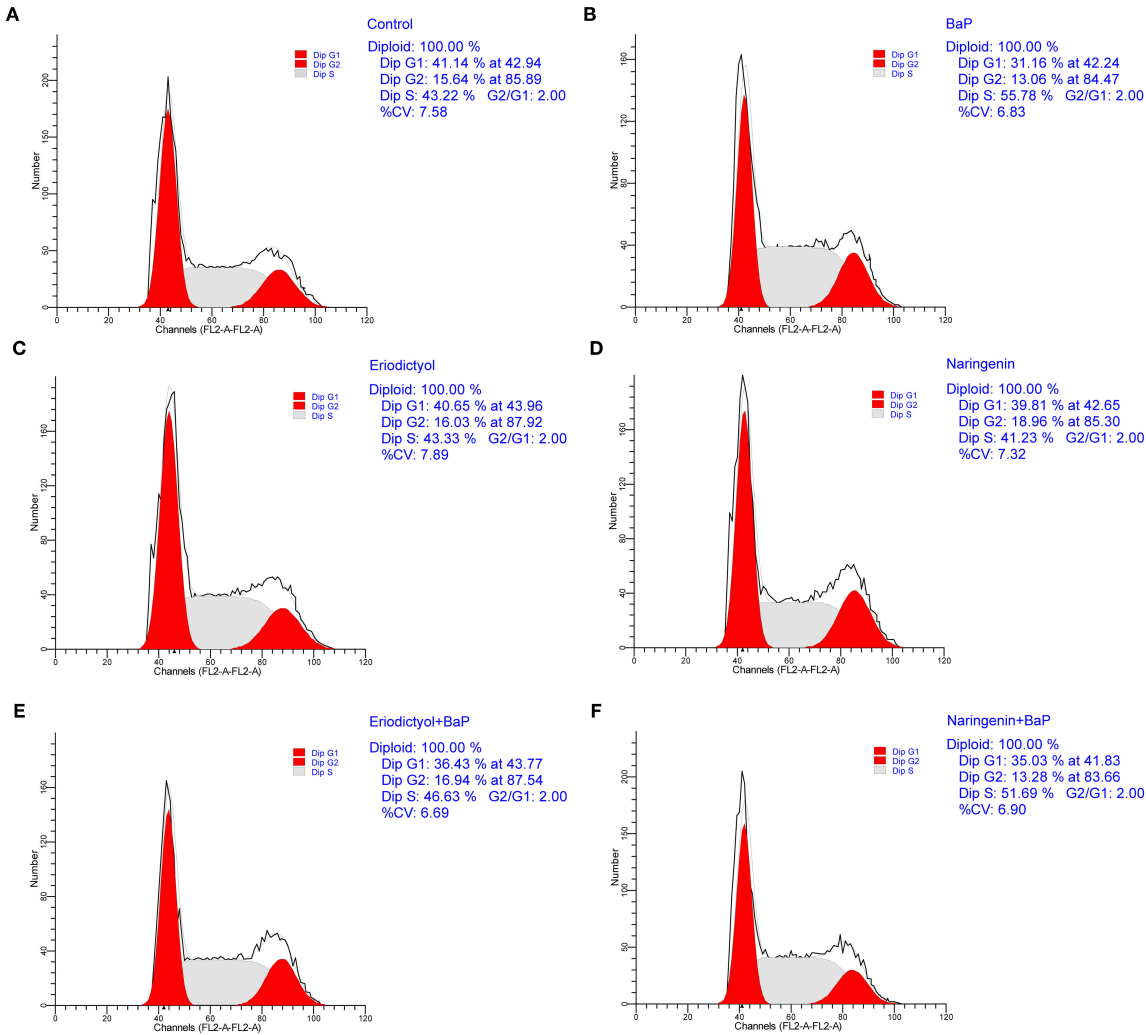
Effect of eriodictyol and naringenin on BaP-induced cell cycle perturbation. Caco-2 cells were incubated with DMSO (Control) **(A)**, 50 μM BaP **(B)**, 20 μM eriodictyol **(C)**, 20 μM naringenin **(D)**, co-treatment of 20 μM eriodictyol and 50 μM BaP **(E)**, and co-treatment of 20 μM naringenin and 50 μM BaP **(F)** for 24 h. Cells were stained with propidium iodide (PI) and analyzed for DNA by flow cytometry. Horizontal and vertical axes indicate the relative nuclear DNA content and number of cells.

To ascertain whether BaP inhibited growth of Caco-2 cells *via* perturbation of the cell cycle, DNA contents were determined using flow cytometry (Figure 2; the statistical analyses are shown in Supplementary Figure 1). Compared to control cells, there were no significant differences in cell cycle parameters when cells were exposed to eriodictyol and naringenin alone. Treatment with BaP for 24 h significantly decreased the percentage of cells in the G1 phase (31.16%) when compared to the control group (41.14%). The percentage of cells in the G2 phase of cells treated with BaP did not differ from that of control cells. However, the percentage of cells in the G2 phase in cells treated with BaP was lower than that of cells treated with naringenin alone or BaP co-treated with eriodictyol, but overall the changes in the percentage of cells in the G2 phase were modest. Noteworthy, treatment with BaP significantly increased the percentage of cells in the S-phase from 43.22 to 55.78%, and this increase was counteracted by co-treatment with naringenin or eriodictyol. These results indicated that treatment with BaP impaired progression through the S-phase and this inhibition was prevented by the co-treatment with the polyphenols. These results are consistent with other studies. Thus, upon treatment of HT-29 cells with 25 μM BaP, the number of cells in the S-phase increased, concomitantly with a decline in the number of cells in the G1 phase (28). When administrated together with BaP, eriodictyol and naringenin at least partly counteracted the changes of the cell cycle caused by BaP. Thus, the percentage of cells arrested in G1 and S-phase decreased from 46.63% in cell treated with BaP to 36.43% in cell co-treated with eriodictyol, whereas co-treatment with naringenin did not significantly reduce the number of cell in the S-phase. These results are consistent with the study by Liu et al. (29) showing that eriodictyol inhibited epidermal growth factor-induced cell S-phase accumulation and increased the percentage of G1-phase cells.

**Figure 3:**
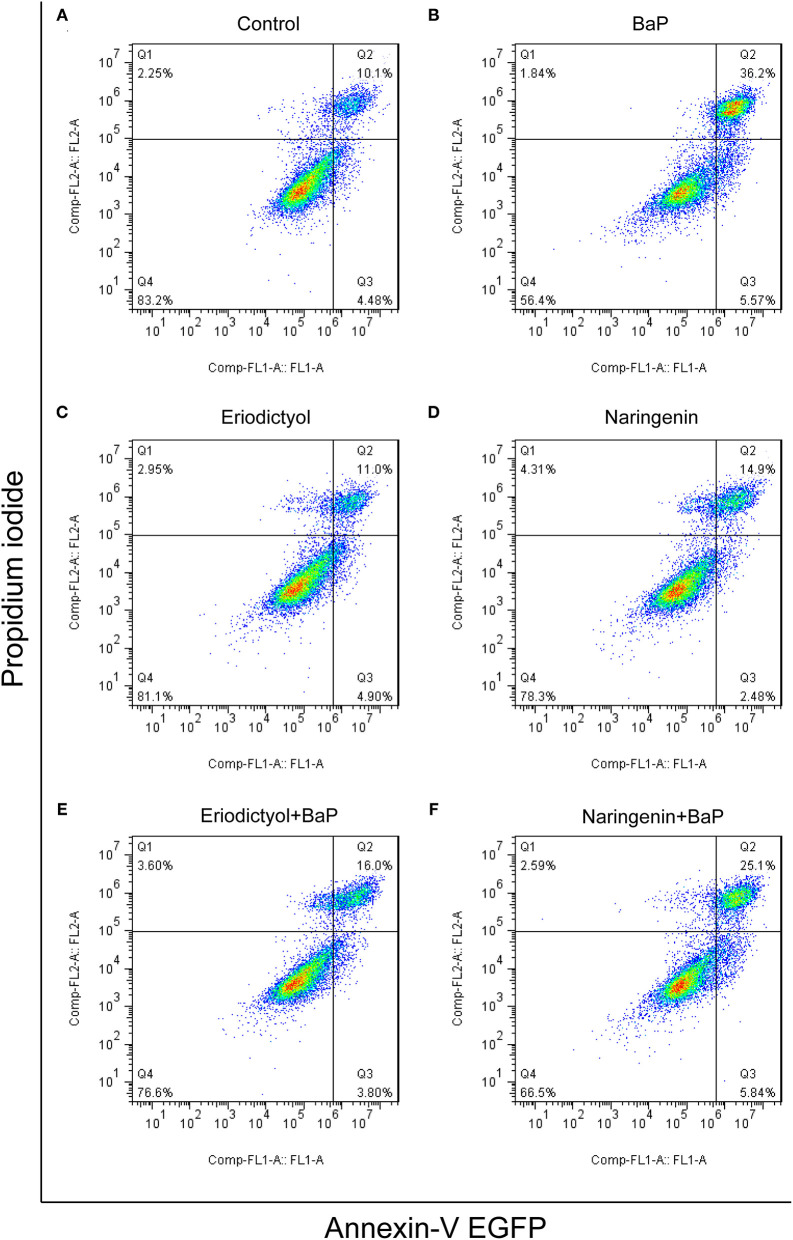
Effect of eriodictyol and naringenin on BaP-induced cell apoptosis. Caco-2 cells were incubated with DMSO (Control) **(A)**, 50 μM BaP **(B)**, 20 μM eriodictyol **(C)**, 20 μM naringenin **(D)**, co-treatment of 20 μM eriodictyol and 50 μM BaP **(E)**, and co-treatment of 20 μM naringenin and 50 μM BaP **(F)** for 24 h. Cell apoptosis was determined by the Annexin-V EGFP/PI assay and flow cytometry.

BaP has been shown to induce apoptosis in primary cultured neurons (6), HL-7702 human normal liver cells (21), and rat lung epithelial cells (30). Metabolism of BaP leads to the formation of BPDE, which can bind to DNA to form BPDE-DNA adducts that may lead to cell apoptosis (31). To assess to what extent BaP induced apoptosis of Caco-2 cells, Annexin-V EGFP and PI staining assay and flow cytometry were used. The results are displayed in Figure 3 (the statistical analyses are shown in Supplementary Figure 2). Based on the report by Wang et al. (32), the cell populations were divided into four regions: the necrotic cells in the Q1 region, cells in the later stage of apoptosis in the Q2 region, cells in the early stage of apoptosis in the Q3 region, and viable cells in the Q4 region. The percentage of cells in the later stage of apoptosis was significantly increased after BaP treatment (36.2%) compared to control cells (10.1%), while eriodictyol treatment alone did not significantly affect the percentage of cells in the stage of later apoptosis.

Of note, co-treatment with eriodictyol or naringenin significantly reduced the percentage of cells in the later stage of apoptosis cells (from 36.2 to 16.0% and 25.1% for eriodictyol and naringenin, respectively). These results were consistent with a previous report showing that eriodictyol was able to protect retinal ganglion cells from high glucose induced oxidative stress and cell apoptosis (33). Another study demonstrated that naringenin reduced apoptosis and oxidative stress in cortical neuron cells (34). The protective effect of eriodictyol seemed superior to that of naringenin. This may be associated with the structure-activity relationship of polyphenols. A previous study demonstrated that catechins and tannins are highly effective in inhibiting BPDE-DNA adduct formation due to direct interaction *via* adjacent hydroxyl groups in their structures and that the activity increases with an increasing number of functional hydroxyl groups (35). In the molecular structure of eriodictyol, there is one more functional hydroxyl group in the B ring than in naringenin.

### Effect of Eriodictyol and Naringenin on BaP-Induced ROS, MDA and SOD Activity

**Figure 4:**
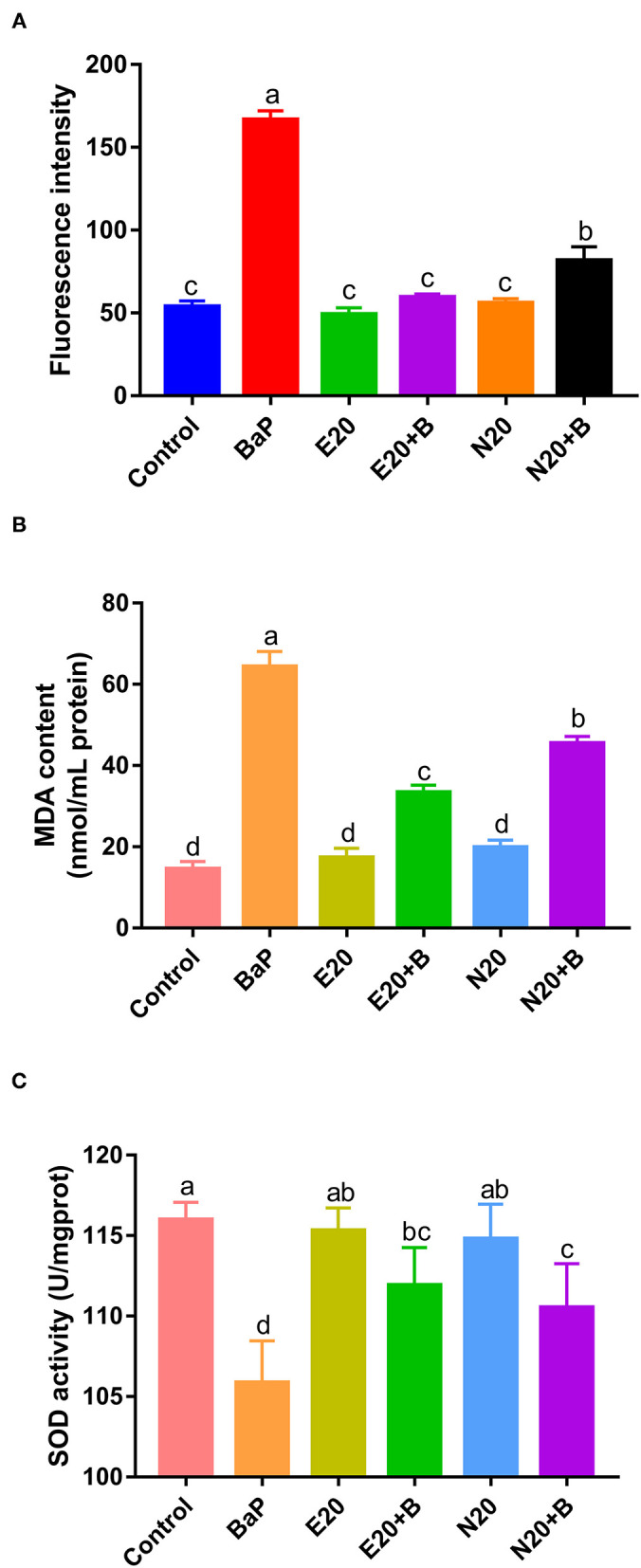
Effect of eriodictyol and naringenin on BaP-induced reactive oxygen species (ROS) **(A)**, MDA content **(B)** and superoxide dismutase (SOD) activity **(C)**. Caco-2 cells were incubated with DMSO (Control), 50 μM BaP, 20 μM eriodictyol, 20 μM naringenin, co-treatment of 20 μM eriodictyol and 50 μM BaP, and co-treatment of 20 μM naringenin and 50 μM BaP for 24 h. ROS was detected by fluorescence microscopy. Error bars represent the standard deviation obtained from three replicated experiments. Bars with different letters are significantly different at the level *p* < 0.05. E refers to eriodictyol, *N* refers to naringenin and B refers to BaP.

BaP has previously been reported to induce oxidative stress *in vivo* and in cultured cells (6, 8). To assess BaP-induced oxidative stress in Caco-2 cells, the induction of oxidative stress markers including intracellular ROS, MDA and SOD was monitored. We used 2′-7′dichlorofluorescein for estimating the level of ROS in Caco-2 cells exposed to BaP and co-treated or not with eriodictyol or naringenin (Figure 4A; Supplementary Figure 3). 2′-7′dichlorofluorescein (DCF) derivatives are relatively nonselective probes that react with many oxidants such as peroxynitrite, hydroxyl radicals, lipid peroxides, nitric oxide, and hypochloride, but not directly with H_2_O_2_ (19, 36). DCF fluorescence is thus a measure of generalized oxidant production rather than that of any particular reactive species. In our study, we aimed to detect BaP-induced generalized oxidation products. In addition, studies have reported that intracellular ROS of cells treated with BaP might be the cause of 8-OHdG formation (37). Here, the aim was to monitor possible signs of ROS production in cells in response to BaP exposure. Therefore, in keeping with a vast number of studies we chose to use the 2′-7′dichlorofluorescein protocol in our study. Thus, we observed an increase in the number of fluorescent cells and fluorescence intensity upon treatment with BaP compared to the control group. Co-treatment with eriodictyol or naringenin significantly reduced the number of fluorescent cells and the fluorescence intensity (Figure 4A; Supplementary Figure 3). The cellular content of MDA was significantly increased when cells were treated with BaP (Figure 4B), whereas the SOD activity markedly decreased (Figure 4C). For both MDA and SOD, co-treatment with eriodictyol or naringenin partly restored the level toward that of the control cells. In BaP-treated Caco-2 cells, the cell viability was significantly reduced compared with control cells, possibly reflecting an imbalance between oxidation and antioxidant systems caused by the massive ROS accumulation and impaired ROS-scavenging capacity (22).

A previous study similarly demonstrated that eriodictyol and naringenin suppressed oxidative stress and apoptosis in cells (33), and that treatment with naringenin up-regulated SOD activity and reduced the levels of MDA and ROS (34). These results further supported the protective effects of eriodictyol and naringenin on alleviation of BaP-induced cell damage. Again we observed that the protective effect of eriodictyol was superior to that of naringenin. Therefore, in order to further investigate possible mechanisms, we used the simultaneous treatment of eriodictyol and BaP, coupled with proteomics analyses for follow-up studies.

### Comparative Proteomic Analysis of the Effect of Eriodictyol on BaP-Induced Cytotoxicity

**Figure 5:**
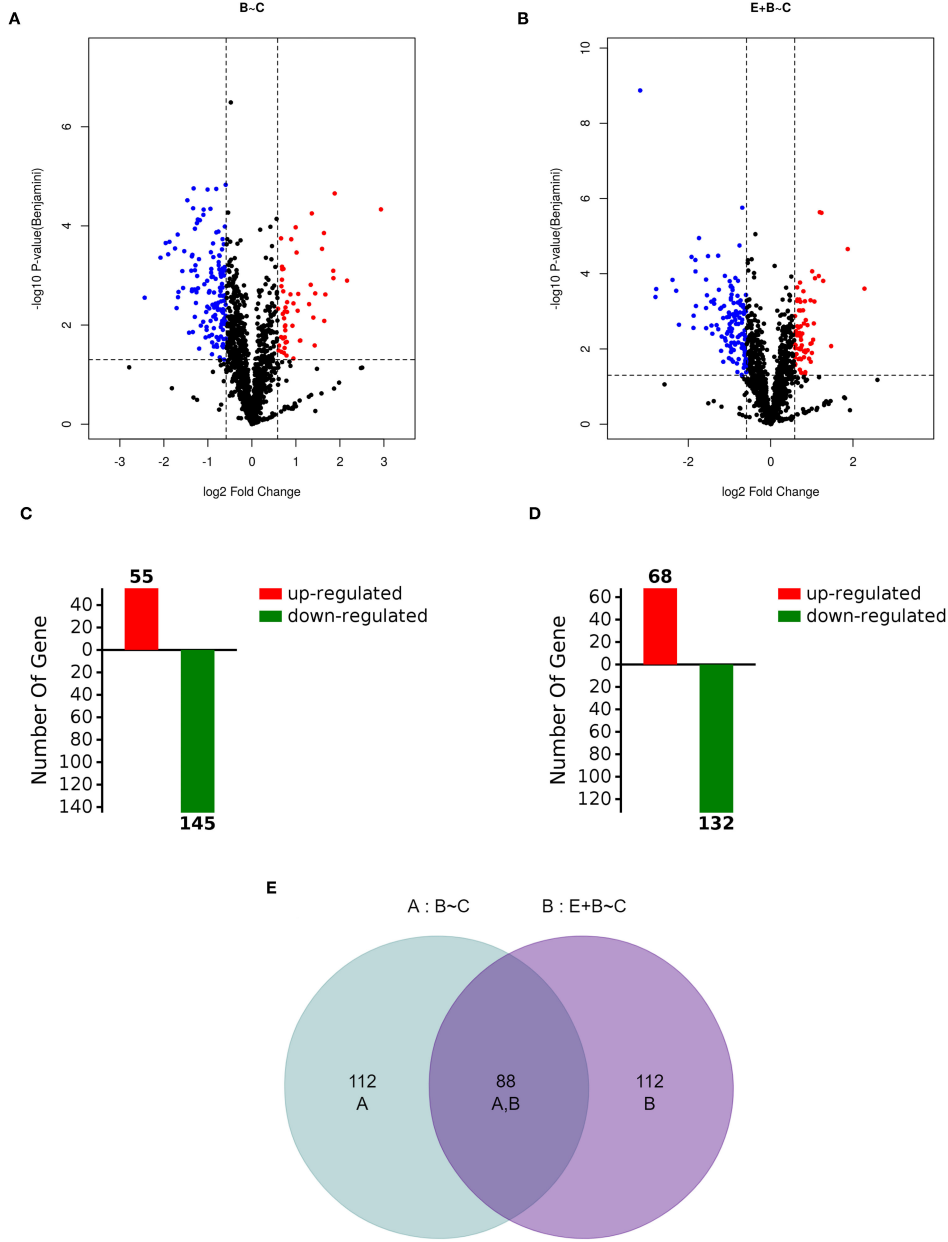
Volcano plot of BaP/Control comparison group (B~C, **A**) and Eriodictyol+BaP/Control comparison group (E+B~C, **B**), expression profiles of BaP/Control comparison group **(C)** and Eriodictyol+BaP/Control comparison group **(D)** and Venn diagrams **(E)** of identified differentially expressed proteins (DEPs). Caco-2 cells were incubated with DMSO (Control), 50 μM BaP, 20 μM eriodictyol and co-treatment of 20 μM eriodictyol and 50 μM BaP for 24 h. Proteins were identified using label-free and LC-MS/MS.

Mass spectrometry-based label-free quantitative techniques were applied to determine the proteomic profiles of Caco-2 cells treated with DMSO (control, C), BaP alone (B) and co-treated with eriodictyol and BaP (E+B). A total of 1571 proteins were identified in the treatment groups including 200 differentially expressed proteins (DEPs) with an average fold change in intensity [BaP/Control (B/C) or Eriodictyol+BaP/Control (E+B/C)] ≥ 1.5 or ≤ 0.67, and a *p* value <0.05 (Figure 5; Supplementary Table 1). Among these DEPs, 55 and 68 of the DEPs were up-regulated, and 145 and 132 of DEPs were down-regulated in the B/C and the E+B/C groups, respectively (Figures 5C,D). Of these, 88 DEPs were shared between the two groups (Figure 5E).

**Figure 6:**
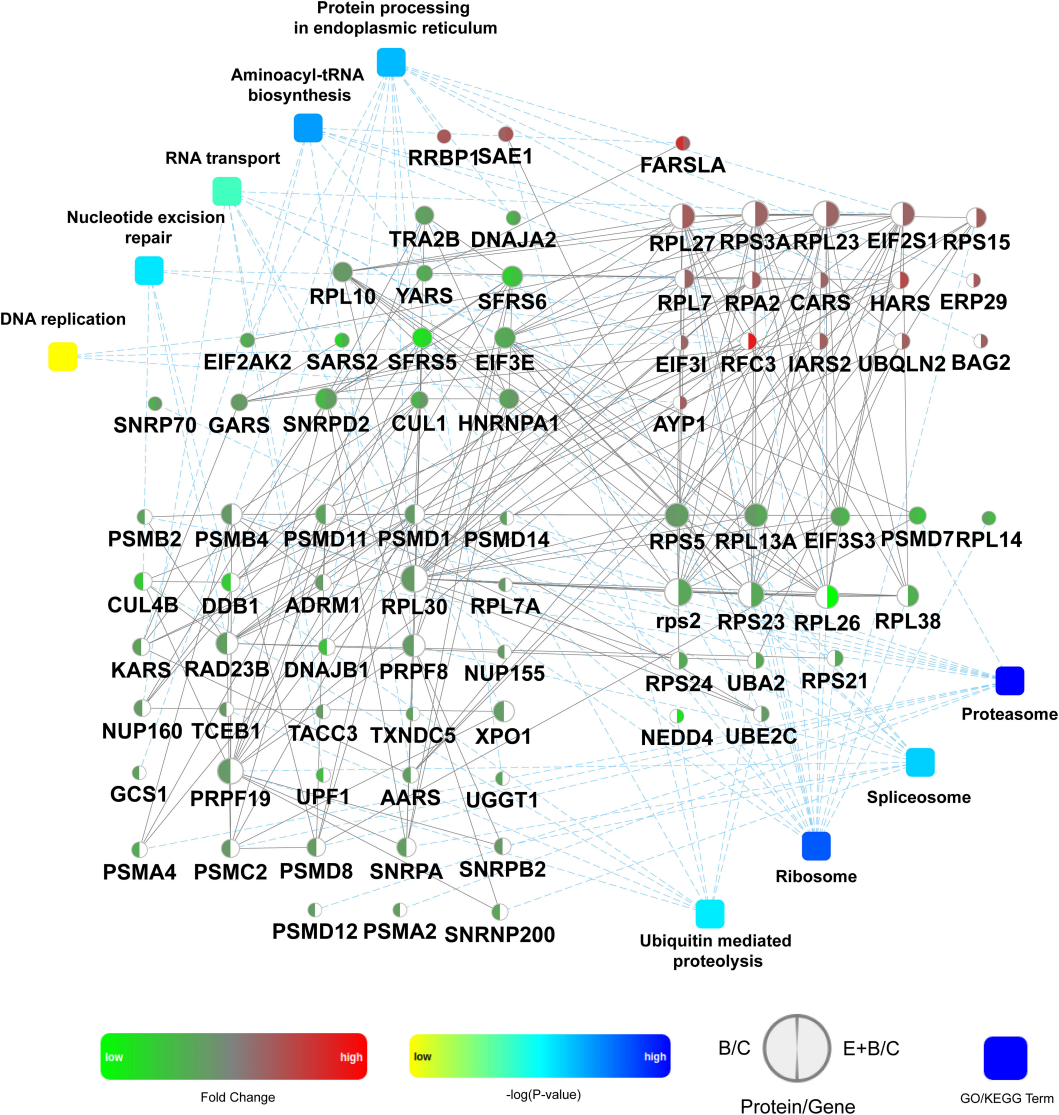
Protein-protein interactions analysis of 80 differentially expressed proteins (DEPs) involved in genetic information processing. The network model was generated by Omicsbean. Circle nodes for genes/proteins, rectangle for KEGG pathway or biological process. Pathways were colored with gradient color from yellow to blue, yellow for smaller *P*-value, and blue for larger *P*-value. In case of fold change analysis, genes/proteins exhibiting significant changes in expression are colored in red (up-regulation) and green (down-regulation) and no significant expression genes/proteins are colored in white. A default confidence cutoff of 400 was used: interactions with bigger confident scores are show as solid lines between genes/proteins, otherwise as dashed lines.

Subsequently, the identified DEPs were distributed into 4 categories based on KEGG annotation comprising metabolism, genetic information processing, cellular processes, and organismal systems (Supplementary Figure 4). Of note, 80 DEPs associated with genetic information processing were identified, representing the highest proportion and number of identified DEPs. The expression of these 80 DEPs in the B/C and the E+B/C treatment groups based on protein-protein interactions (PPI) was further analyzed (Figure 6). Some proteins are involved in more than one pathway. For instance, RFC3 participates in the processes of DNA replication and nucleotide excision repair (NER) explaining why there are 88 DEPs involved in genetic information processing in Supplementary Figure 4, while only 80 were displayed in the PPI network.

DNA replication was enriched into the genetic information processing category (Figure 6). A previous study reported that BaP metabolites bound to DNA may interfere with the vital cellular process of DNA replication, leading to an accumulation of mutations and eventually carcinogenesis (11). It has been reported that RPA may control DNA repair and damage checkpoint activation in this pathway (38). Our results showed that RPA2 was up-regulated in the E+B/C group, with no significant change in the B/C group, indicating that eriodictyol prevents BaP-induced cytotoxicity by activating the expression of RPA2 to repair damaged DNA. Moreover, BaP-DNA adducts can be removed by NER (39). In this study, the expression of CUL4B, DDB1, RAD23B involved in NER was down-regulated after BaP treatment, which may result in a deficiency of these proteins. Of note, the expression of these three proteins was recovered in the E+B/C group. Furthermore, since the expression of RPA2 and RFC3 involved in NER was up-regulated after co-treatment, we speculate that they may play a central role in restoring the NER process. SNRPA is a component of the spliceosomal U1 small nuclear ribonucleoprotein (snRNP), which is essential for recognition of the pre-mRNA 5' splice-site and the subsequent assembly of the spliceosome (40). Export of mRNAs through the nuclear pore complex (NPC) from the nucleus to the cytoplasm is a key regulatory step in the expression of proteins (41). In our study, SNRPA and NUP155 were down-regulated after BaP treatment, indicating that BaP may affect mRNA processing and export impairing central cell biological processes. In addition, a possibly impairment of aminoacyl-tRNA biosynthesis was observed as indicated by down-regulation of AARS, KARS, YARS, GARS and SARS2 in the B/C group.

Proteasomes are large protein complexes and play roles in apoptosis and cell cycle regulation (42). We observed that the level of all identified proteins involved in proteasomes was down-regulated in the B/C group, indicating that they might be associated with the significant changes in apoptosis and cell cycle progression after BaP treatment. The expression of the proteins involved in aminoacyl-tRNA biosynthesis and proteasomes did not significantly differ between co-treated and control cells.

### Confirmation of Altered Expression of Selected Proteins

**Figure 7:**
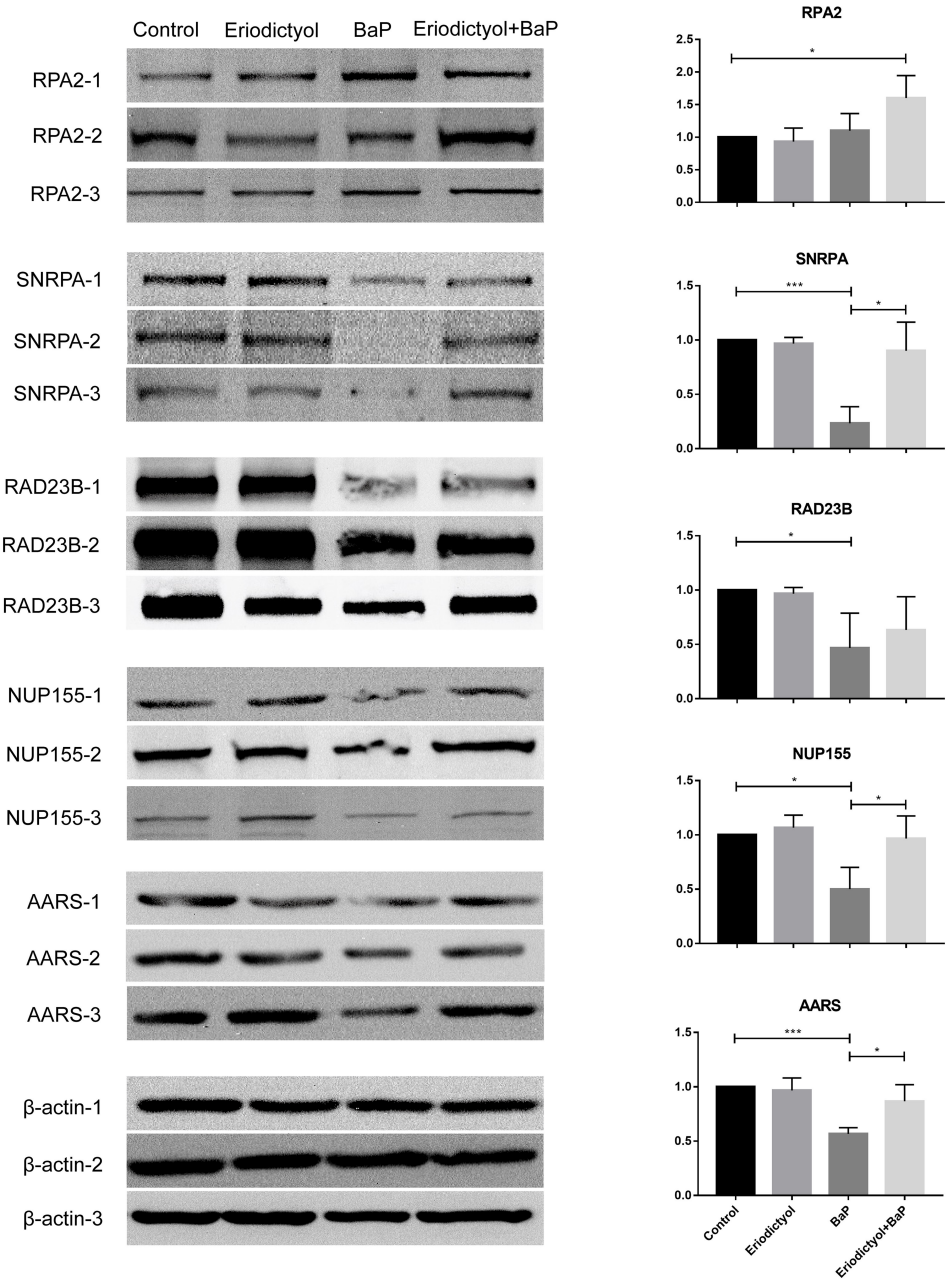
Analysis of expression of selected proteins by western blotting. Caco-2 cells were treated with DMSO (control), 50 μM BaP, 20 μM eriodictyol and co-treatment of 20 μM eriodictyol and 50 μM BaP for 24 h. *p < 0.05, **p < 0.01, and ***p < 0.001.

To confirm the expression of selected proteins based on proteomics, Western blotting and statistical analyses were performed (Figure 7). The expression of SNRPA, RAD23B, NUP155 and AARS was down-regulated in cells treated with BaP, whereas eriodictyol treatment at least partly restored the expression of these proteins, implying that eriodictyol may play a protective role. In addition, the expression of RPA2 was up-regulated after co-treatment treatment, while its expression did not be changed in the BaP treatment group. These results were in agreement with the proteomics data. In addition, previous studies have reported similar results, with similar expression trends of RPA2, SNRP31 and RAD23B observed after BaP exposure (43, 44). Interestingly, the proteins reported in previous studies are all involved in the process of transcription, while in the present study, we found that specific proteins involved in the translation process also presented significant changes, which indicates that BaP might affect several central biological processes.

**Figure 8:**
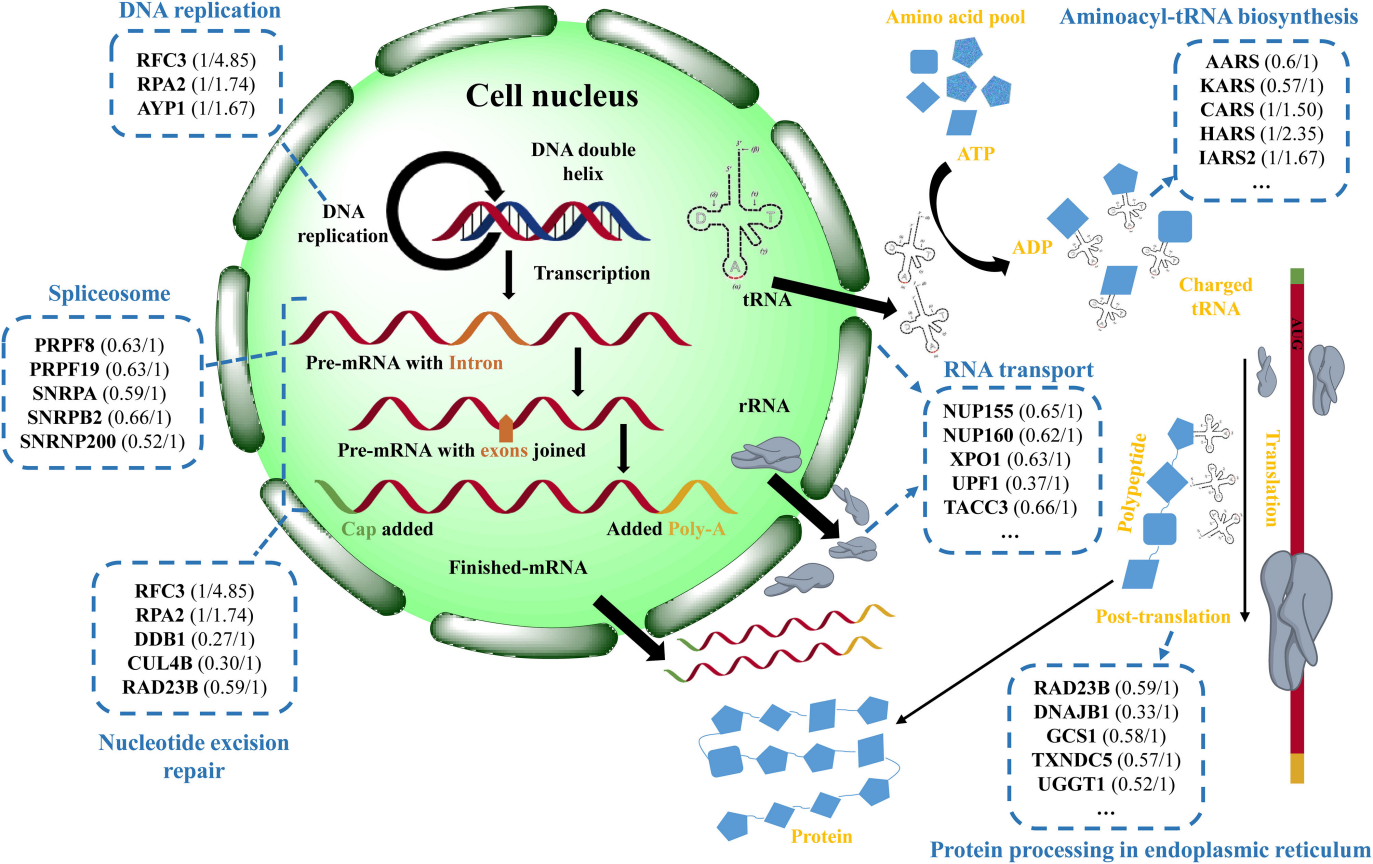
Schematic presentation of the potential protective effect of eriodictyol on BaP-induced cytotoxicity. The figure shows the changes of differentially expressed proteins (DEPs) in cells treated with BaP or eriodictyol+BaP classified according to their involvement in different biological processes. The names of the corresponding representative DEPs and expression profiles (the left is the fold change of BaP/control and the right is the fold change of eriodictyol+BaP/control) in each process are shown in the blue dashed-lined boxes. DEPs are filtered with an average fold change in intensity ≥ 1.5 or ≤ 0.67, and a *p* value <0.05.

Based on the above results and analyses, a possible protective mechanism of eriodictyol alleviating cytotoxicity induced by BaP is summarized in Figure 8, i.e., eriodictyol plays a protective role by regulating the expression of key proteins in transcription and translation. Other identified proteins may also make an important contribution in the cellular response to BaP, and further studies of these proteins may provide a better understanding of the mechanisms of BaP-induced Caco-2 cytotoxicity.

## Conclusion

In this study, our results showed that eriodictyol and naringenin have a protective effect on BaP induced cell apoptosis, cell cycle progression, and oxidative stress, especially for eriodictyol. A total of 80 differentially expressed proteins (DEPs) were identified in response to treatment with eriodictyol. Proteins associated with genetic information processing pathways represented the highest proportion amongst the DEPs and included key proteins such as RPA2, SNRPA, RAD23B, NUP155 and AARS. These results provided new insights into the role of polyphenol in inhibiting BaP-induced cell damage, not only through the DNA replication pathway. In addition, treatment with quinic acid, ferulic acid, homovanillic acid and trolox decreased cell viability, indicating that a potential adverse effect of polyphenols should be considered when choosing suitable chemical protective agents.

All claims expressed in this article are solely those of the authors and do not necessarily represent those of their affiliated organizations, or those of the publisher, the editors and the reviewers. Any product that may be evaluated in this article, or claim that may be made by its manufacturer, is not guaranteed or endorsed by the publisher.

## Data Availability Statement

The datasets presented in this study can be found in online repositories. The names of the repository/repositories and accession number(s) can be found below: http://www.proteomexchange.org/, PXD030049.

## Author Contributions

CW and GZ designed and conceived the research. CW and FZ drafted the manuscript. CW, FZ, YB, and XX analyzed the data and interpreted the results. CL and KK reviewed and extensively edited the final manuscript. All authors contributed to the article and approved the submitted version.

## Funding

This research was supported by the National Natural Science Foundation of China (32001721) and Priority Academic Program Development of Jiangsu Higher Education Institutions (RAPD).

## Conflict of Interest

The authors declare that the research was conducted in the absence of any commercial or financial relationships that could be construed as a potential conflict of interest.

## Publisher's Note

All claims expressed in this article are solely those of the authors and do not necessarily represent those of their affiliated organizations, or those of the publisher, the editors and the reviewers. Any product that may be evaluated in this article, or claim that may be made by its manufacturer, is not guaranteed or endorsed by the publisher.

## Supplementary Material

Supplementary Figure 1 Statistical analyses of cell cycle perturbation. Bars with different letters are significantly different at the level *p* < 0.05.

Supplementary Figure 2 Statistical analyses of cell apoptosis. Bars with different letters are significantly different at the level *p* < 0.05.

Supplementary Figure 3 Fuorescent images of intracellular ROS in Caco-2 cells co-treated with eriodictyol/naringenin and BaP.

Supplementary Figure 4 Classes of enriched KEGG Pathways. The general information of the enrichment includes pathway name, *p* value (calculated with Fisher's exact test with Hypergeometric algorithm), count (number of genes/proteins in the query that are involved in this term).

Supplementary Figure 5 The QC analysis for the label-free method. Basepeak of chromatograms of Caco-2 cells treated with BaP **(A)**, DMSO **(B)**, and BaP and eriodictyol **(C)**. Correlation analysis **(D)** and normal distribution diagrams **(E)** of protein intensity in different samples.

Supplementary Table 1 Summary information of the identified differentially expressed proteins (DEPs).
