# Supplementary figures and images for: Proteomic Analysis of the Protective Effect of Eriodictyol on Benzo(a)pyrene-Induced Caco-2 Cytotoxicity
Source: Front Nutr. 2022 Mar 3;9:839364. doi: 10.3389/fnut.2022.839364 (PMC8927910; doi:10.3389/fnut.2022.839364)

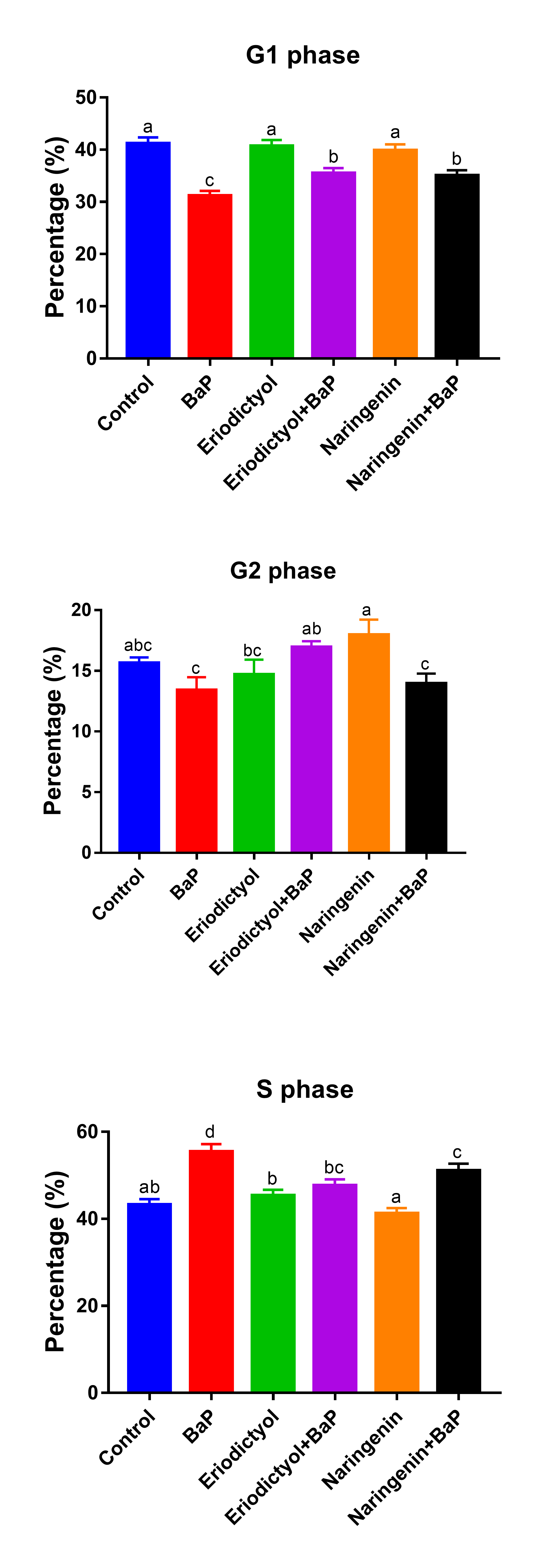

Supplement: Supplementary Figure 1 — Statistical analyses of cell cycle perturbation. Bars with different letters are significantly different at the level p < 0.05. [file Image_1.TIF]

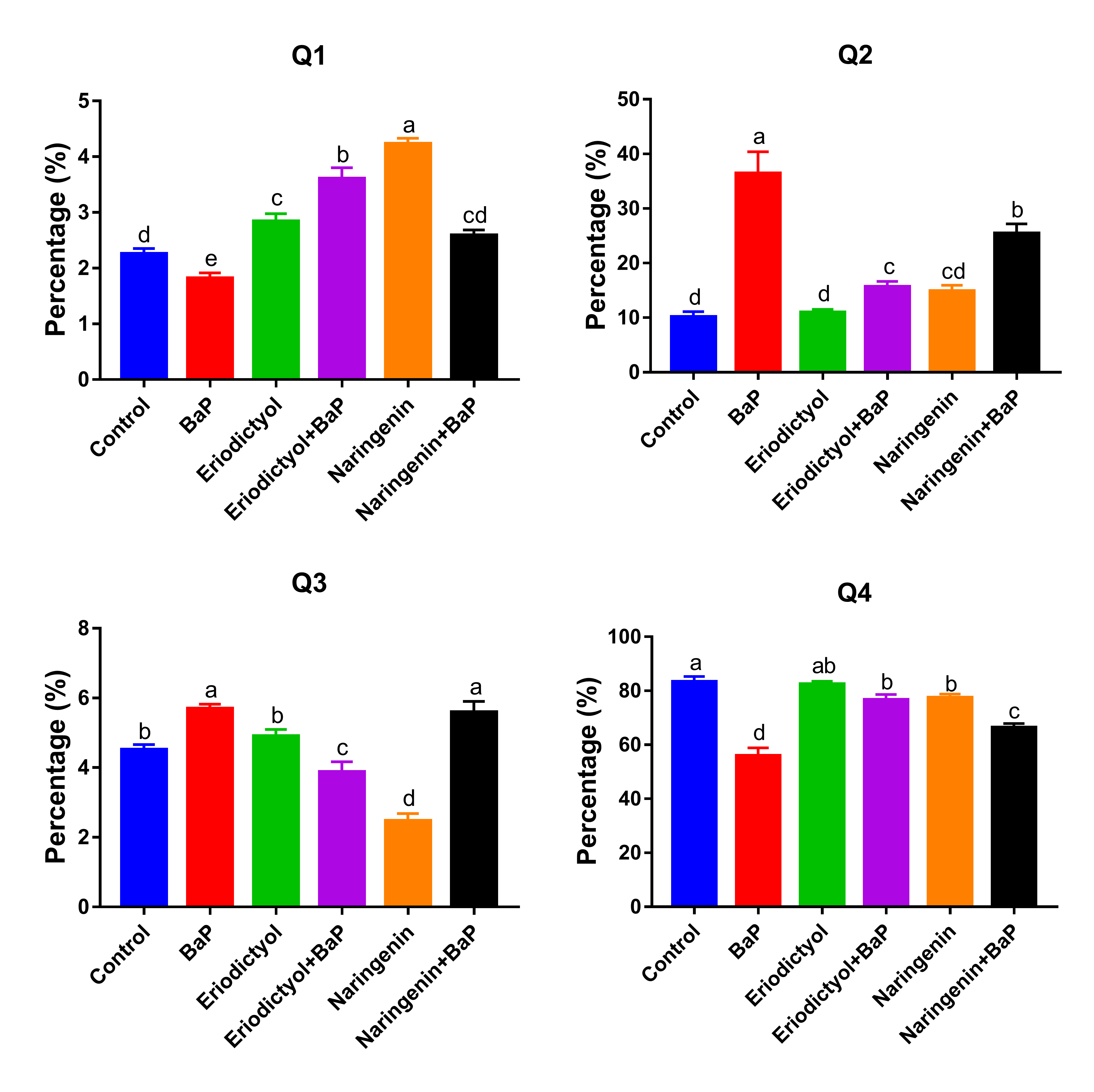

Supplement: Supplementary Figure 2 — Statistical analyses of cell apoptosis. Bars with different letters are significantly different at the level p < 0.05. [file Image_2.TIF]

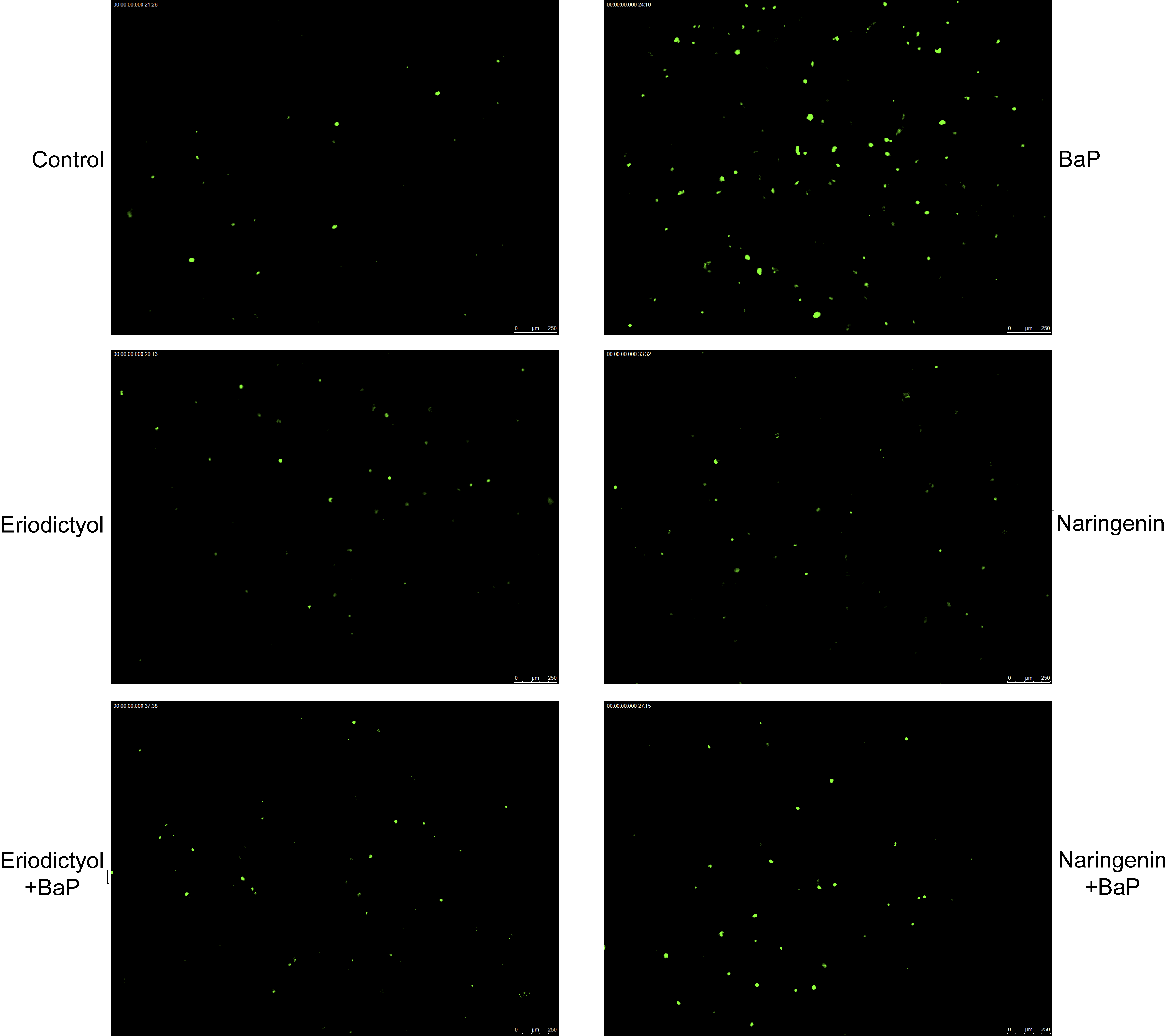

Supplement: Supplementary Figure 3 — Fuorescent images of intracellular ROS in Caco-2 cells co-treated with eriodictyol/naringenin and BaP. [file Image_3.TIF]

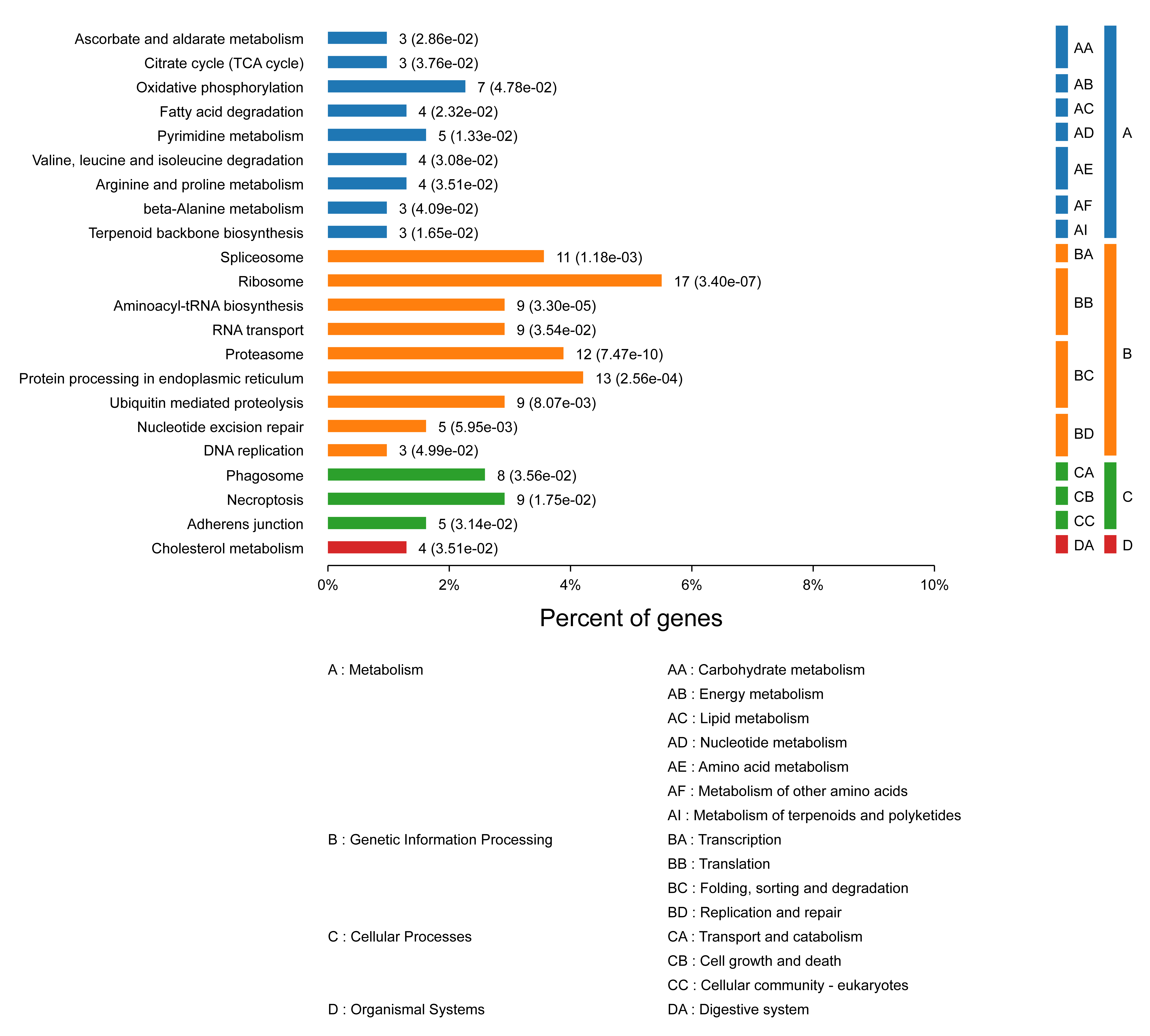

Supplement: Supplementary Figure 4 — Classes of enriched KEGG Pathways. The general information of the enrichment includes pathway name, p value (calculated with Fisher's exact test with Hypergeometric algorithm), count (number of genes/proteins in the query that are involved in this term). [file Image_4.TIF]
