# Supplementary material for: Proteomic Analysis of the Protective Effect of Eriodictyol on Benzo(a)pyrene-Induced Caco-2 Cytotoxicity
Source: Front Nutr. 2022 Mar 3;9:839364. doi: 10.3389/fnut.2022.839364 (PMC8927910; doi:10.3389/fnut.2022.839364)
Supplement: Supplementary Figure 5 — The QC analysis for the label-free method. Basepeak of chromatograms of Caco-2 cells treated with BaP (A), DMSO (B), and BaP and eriodictyol (C). Correlation analysis (D) and normal distribution diagrams (E) of protein intensity in different samples. [file Image_5.pdf]

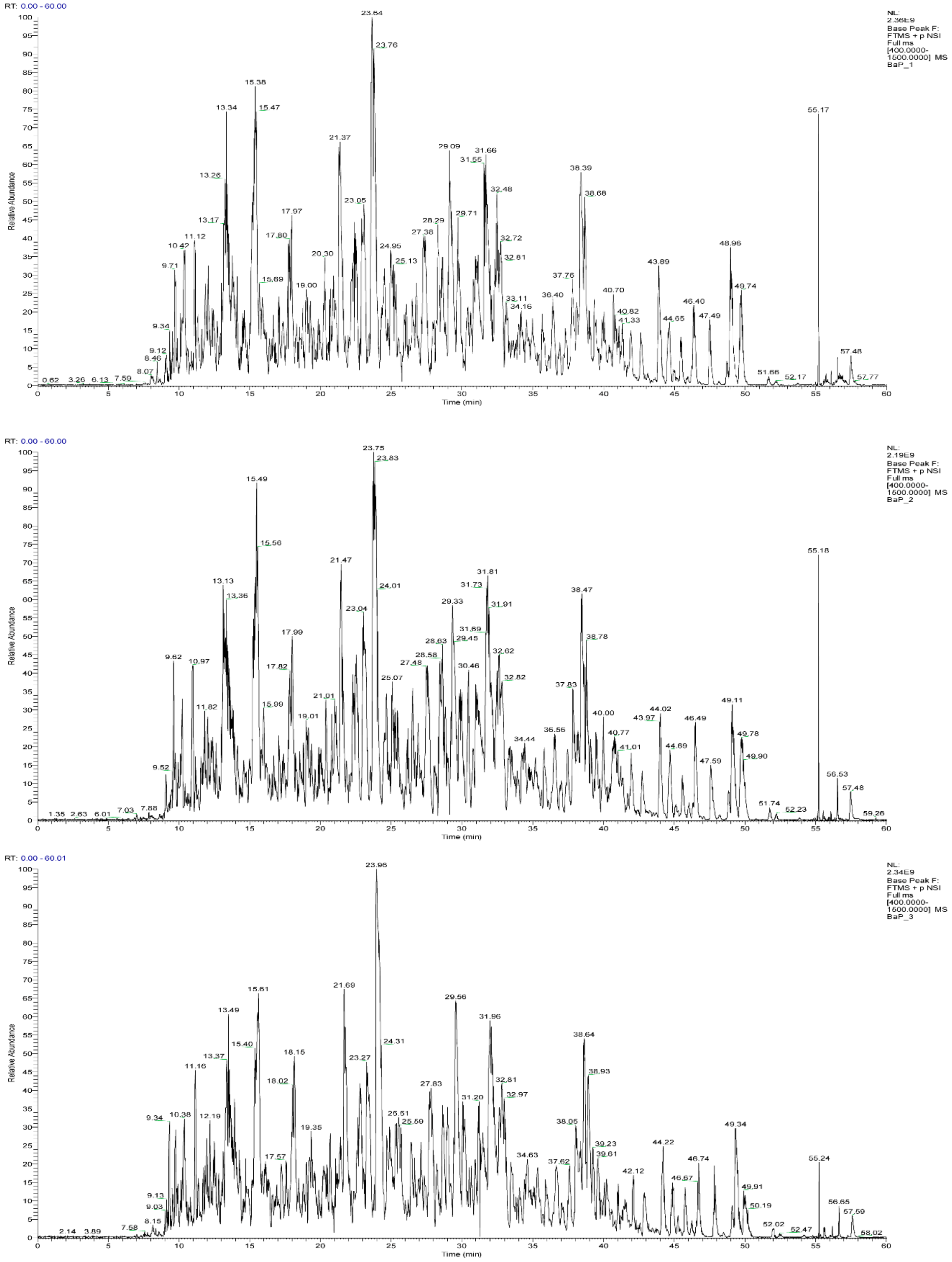

Figure 5A. Basepeak of chromatograms of Caco-2 cells treated with BaP

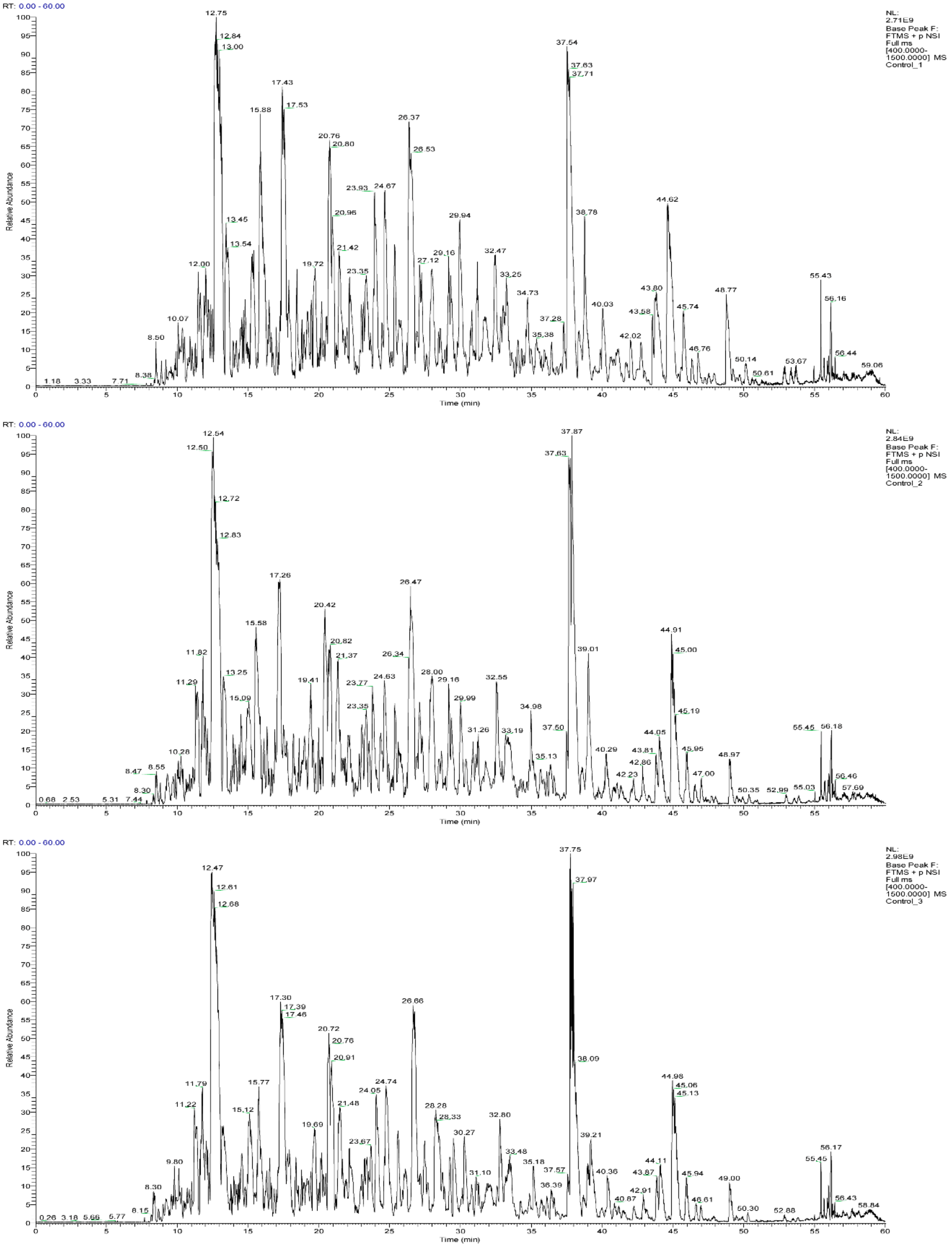

Figure 5B. Basepeak of chromatograms of Caco-2 cells treated with DMSO

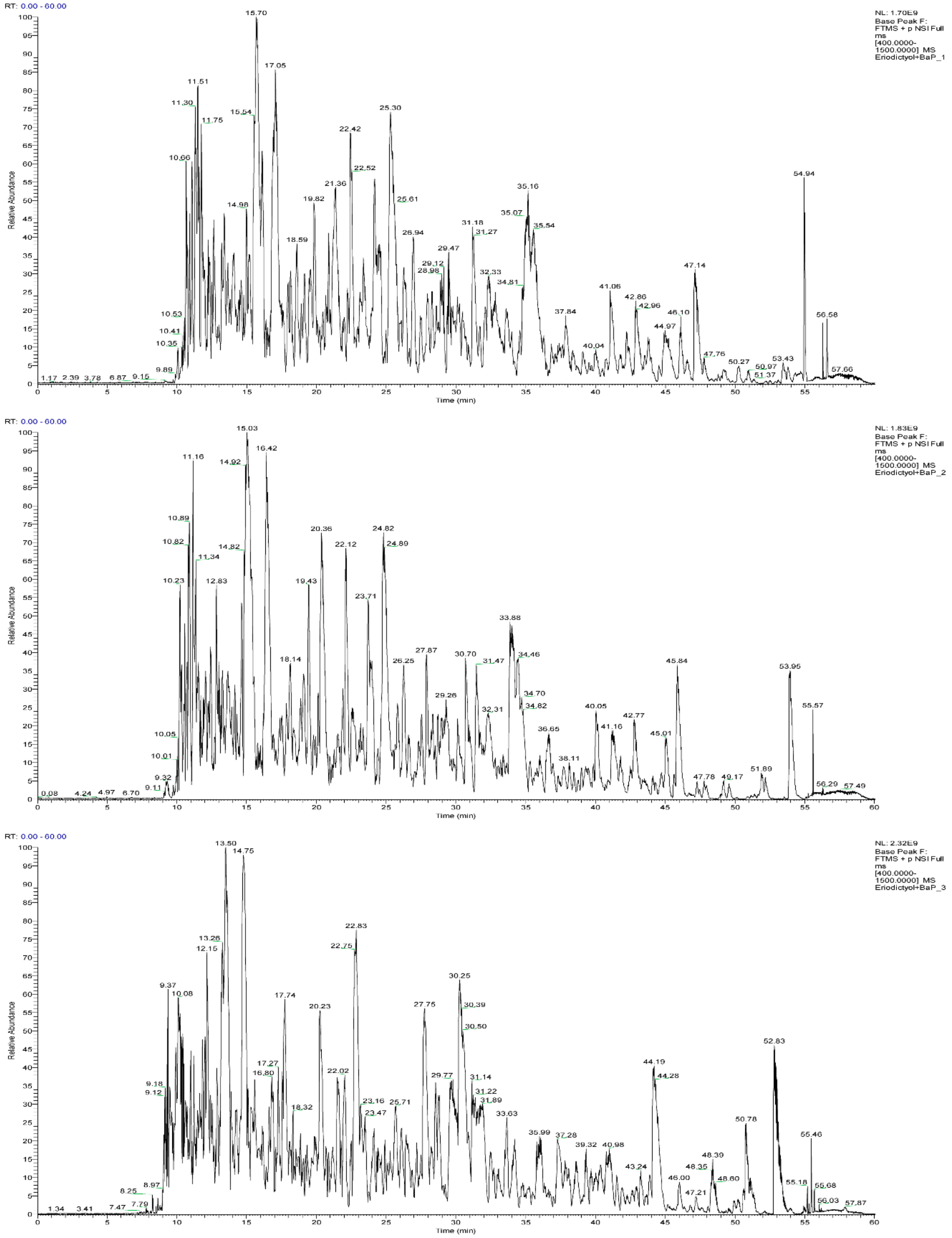

Figure 5C. Basepeak of chromatograms of Caco-2 cells treated with BaP and eriodictyol

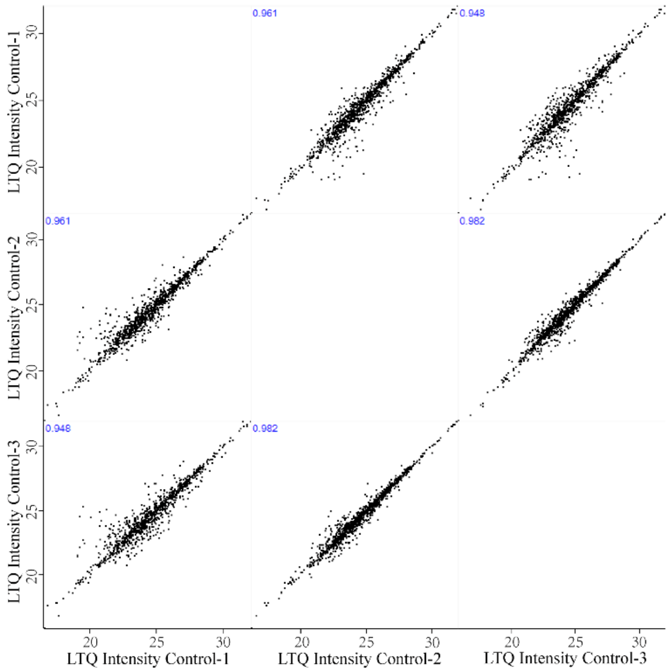
0.961
0.948
0.961
0.982
0.948
0.982
LTQ Intensity Control-1
LTQ Intensity Control-2
LTQ Intensity Control-3
20
25
30
LTQ Intensity Control-1
LTQ Intensity Control-2
LTQ Intensity Control-3

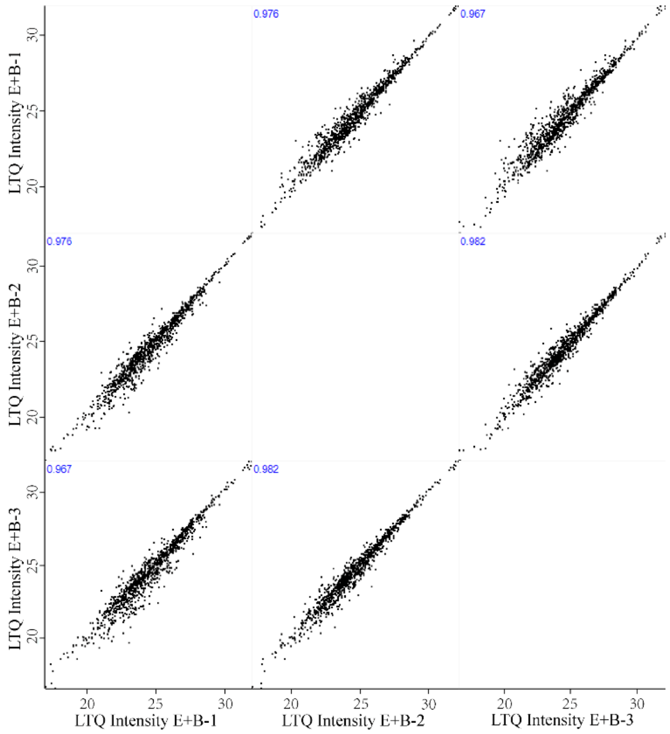
0.976
0.967
0.976
0.982
0.967
0.982
LTQ Intensity E+B-1
LTQ Intensity E+B-2
LTQ Intensity E+B-3
20
25
30
LTQ Intensity E+B-1
LTQ Intensity E+B-2
LTQ Intensity E+B-3

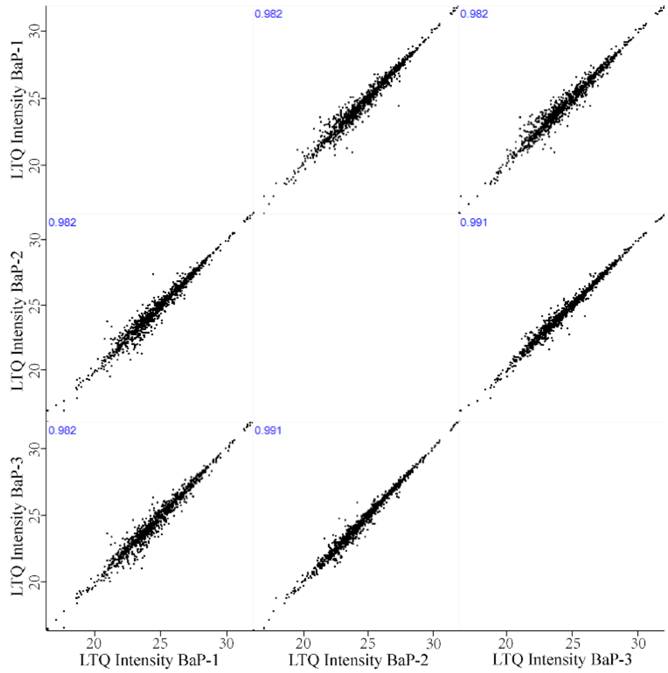

Figure 5D. Correlation analysis of protein intensity in different samples

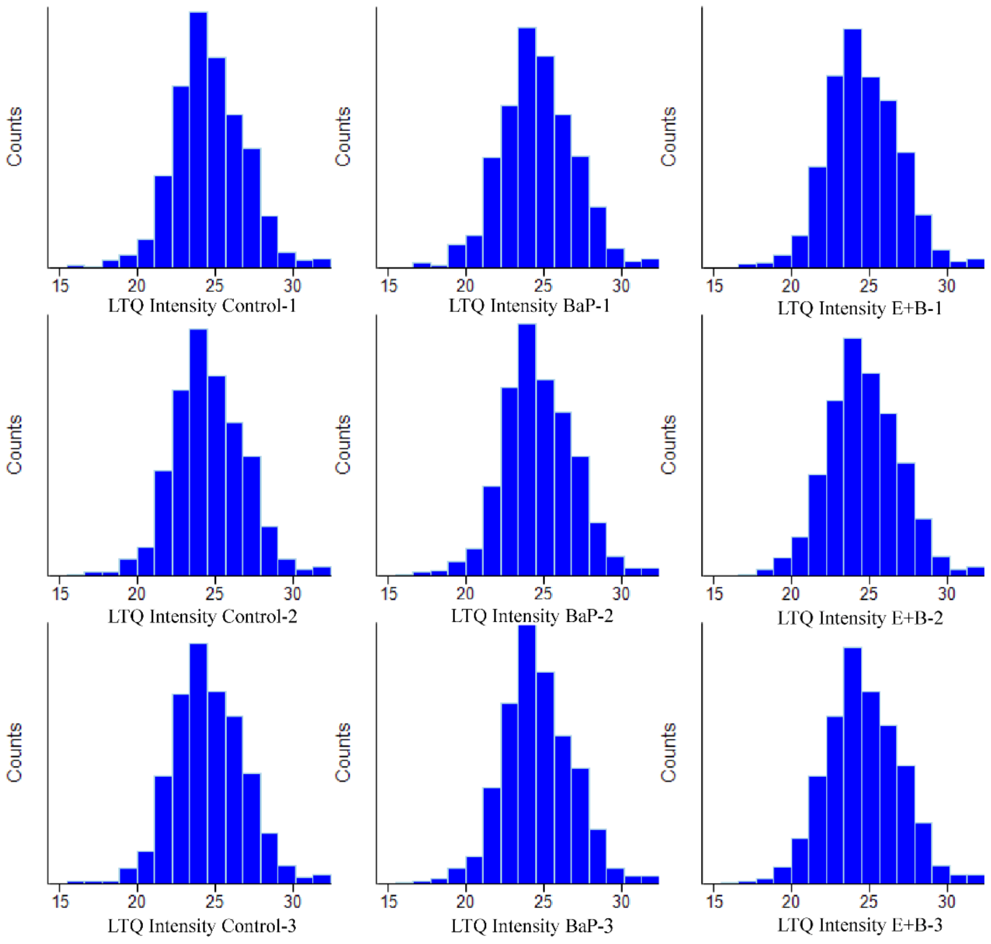

Figure 5E. Normal distribution diagrams of protein intensity in different samples
